# Association between remnant cholesterol and depression in middle-aged and older Chinese adults: a population-based cohort study

**DOI:** 10.3389/fendo.2025.1456370

**Published:** 2025-02-03

**Authors:** Yang Zhou, Yan Lin, Yanhui Yang, Wang Lei, Juan Xu, Yuanzeng Zhu

**Affiliations:** ^1^ Department of Gastrointestinal Surgery, Henan Provincial People’s Hospital, People’s Hospital of Zhengzhou University, People’s Hospital of Henan University, Zhengzhou, China; ^2^ Department of Endocrinology, The People’s Hospital of Danyang, Danyang Hospital of Nantong University, Danyang, Jiangsu, China; ^3^ Department of Cardiology, The Second Medical Center, Chinese PLA General Hospital, Beijing, China; ^4^ Department of Breast Surgery, Henan Provincial People’s Hospital, People’s Hospital of Zhengzhou University, People’s Hospital of Henan University, Zhengzhou, China; ^5^ Department of General Surgery, Affiliated Xiaoshan Hospital, Hangzhou Normal University, Hangzhou, China

**Keywords:** remnant cholesterol, depression, CHARLS, middle-aged, older

## Abstract

**Background:**

The focus on remnant cholesterol (RC) has intensified because of its association with various diseases. In this study, we investigated the association between RC and depression in middle-aged and older adults.

**Methods:**

The study involved 7,305 participants from the 2015 and 2018 waves of the China Health and Retirement Longitudinal Study. Based on the 10-item Center for Epidemiological Studies Depression Scale (CESD-10), depression was indicated by scores ≥ 12. To assess the correlation between RC levels and depression, a logistic regression model that incorporated restricted cubic spline techniques was used.

**Results:**

Of the study population, (mean age: 60.0 ± 9.5 years), 50.3% were female. From 2015 to 2018, the mean CESD-10 score increased from 6.31 ± 3.56 to 7.85 ± 5.23. Following adjustment for confounding factors, individuals in the higher RC level quartile exhibited a higher depression risk (Q3: odds ratio [OR]: 1.75, 95% confidence intervals [CI]: 1.29–2.39; Q4: OR: 2.68, 95% CI: 1.96–3.68, P for trend < 0.001), with a linear correlation between RC levels and depression (P for nonlinearity = 0.108). And the subgroup analysis yielded results consistent with the primary findings.

**Conclusion:**

This study revealed that in China, in middle-aged and older individuals, elevated RC levels were associated with a higher depression risk, suggesting RC is a promising target for depression prevention and treatment.

## 1 Introduction

Depression, a prevalent mood disorder, has significant implications for the overall quality of life as well as potential disability- and mortality-associated outcomes (1–3). In middle-aged and older adults, the propensity to overlook depression and leave it untreated is often attributed to a high comorbidity prevalence, which emphasizes the crucial need for enhanced depression prevention strategies.

Previous studies indicate that depression is associated with elevated total cholesterol (TC) (4) and triglycerides levels (5), as well as reduced low-density lipoprotein cholesterol (LDL-C) (6), high-density lipoprotein cholesterol (HDL-C) (5), and omega-3 polyunsaturated fatty acids levels (7). However, other studies have reported no association between depression and LDL-C (4, 8) or TC levels (8, 9), or found associations only in certain populations (6). The inconsistent results constrain the clinical utility of lipid biomarkers, highlighting the necessity to investigate novel lipid parameters for predicting and intervening in depression.

In fasting and non-fasting conditions, remnant cholesterol (RC), or triglyceride-rich cholesterol, is mainly made up of very low-density lipoproteins, intermediate-density lipoproteins, and chylomicron remnants (10, 11). RC has been associated with various diseases, including metabolic, cardiovascular, and diabetes (12–14). Studies have shown that RC may predict cardiovascular disease comparably or even more reliably than LDL-C or TC (15). Although extensive research has been conducted about the role of RC in cardiovascular disease, there has been limited attention on its role in depression. A recent cross-sectional study reported a potential, significant positive correlation between RC and depression in United States adults (16), highlighting the potential to use RC as a novel, predictive biomarker for depression.

This study, based on data from the China Health and Retirement Longitudinal Study (CHARLS), investigated the relationship between RC and depression in middle-aged and older populations to obtain population-based evidence of the association between RC and depression.

## 2 Materials and methods

### 2.1 Study population

**Figure 1:**
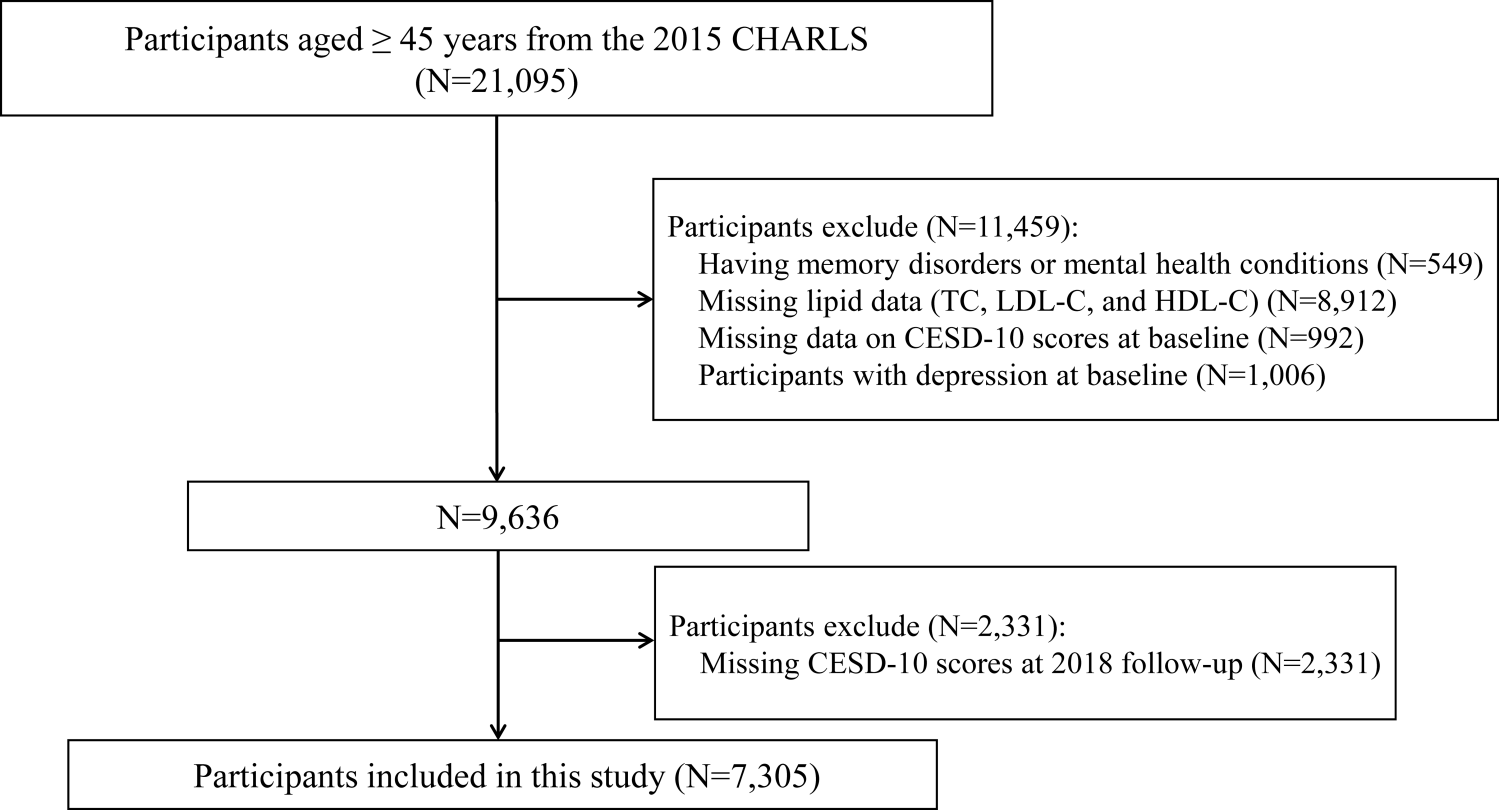
Study flowchart. CHARLS, China Health and Retirement Longitudinal Study; CESD-10, 10-item Center for Epidemiologic Studies Depression Scale; HDL-C, high-density lipoprotein cholesterol; LDL-C, low-density lipoprotein cholesterol; TC, total cholesterol.

CHARLS, which primarily aims to thoroughly assess population aging in China and promote interdisciplinary aging-related studies, is an extensive effort to collect high-quality microdata that accurately represents Chinese households and individuals aged ≥45 (http://charls.pku.edu.cn/) (17). CHARLS is an ongoing survey conducted from 2011 to 2020, with data collected every 2-3 years across five waves. Using a multi-stage stratified probability sampling method, the study includes demographic data from both rural and urban populations. The baseline survey (Wave 1) was conducted in 2011, followed by subsequent waves in 2013 (Wave 2), 2015 (Wave 3), 2018 (Wave 4), and 2020 (Wave 5). In each wave, CHARLS interviewers used computer-assisted personal interviewing techniques and standardized questionnaires to assess health-related factors. Additionally, certain waves included the collection of blood samples and physical measurement data. In this study, we used data from the 2015 and 2018 waves of the CHARLS survey, with the 2015 wave being used as the baseline. Individuals aged 45 or older were initially included in the database. To ensure the accuracy of the data, specific exclusion criteria were applied: (1) participants with diagnosed memory disorders or mental health conditions (n = 549); (2) participants with missing data on TC, LDL-C, and HDL-C (n = 8,912); (3) no 10-item Center for Epidemiological Studies Depression Scale (CESD-10) scores at baseline (n = 992); (4) Participants with depression at baseline (n = 1,006); (5) Missing CESD-10 scores at 2018 follow-up (n = 2,331). Consequently, the study involved 7,305 participants, who met all inclusion criteria (**Figure 1**).

Ethical approval for the study was granted by the Ethics Committee of Peking University (approval number IRB00001052-11015). All participants gave written informed consent.

### 2.2 Remnant cholesterol

Blood lipid levels were quantified using an enzymatic colorimetric test (18). RC levels were determined as follows: TC minus LDL-C minus HDL-C (19). Based on RC quartiles, participants were divided into Q1 (<10.31 mg/dl), Q2 (≥10.31-16.48 mg/dl), Q3 (≥16.48-24.24 mg/dl), and Q4 (≥24.24 mg/dl).

### 2.3 Depression

Depression was diagnosed using the CESD-10, which is widely used for population-based depressive symptom assessment (20). The CESD-10 contains 10 items, such as “bothered by little things,” “felt depressed,” and “could not get going”, the score for each item is between 0 and 3. Thus, the total score ranges from 0 to 30, with higher scores reflecting greater levels of depression. A score of 12 or above is considered to be experiencing depression (21).

### 2.4 Covariates

To address potential confounding effects, various covariates were examined, including age, gender, body mass index (BMI), place of residence (urban or rural), education level (illiterate, elementary school, middle school, or higher), marital status (single or married), smoking (current smoking), drinking (current drinking), diabetes (yes/no), hypertension (yes/no), dyslipidemia (yes/no), chronic comorbidities (0, 1, 2 or more), overall health status (poor, fair, good, very good, or higher), cognitive function, and CESD-10 score at baseline. Cognitive function was assessed using tests of episodic memory and mental sharpness. The cognitive function score was the sum of these two components, ranging from 0 to 21, with higher scores indicating better cognitive performance.

### 2.5 Statistical analysis

Data were summarized using descriptive statistics and reported as mean ± standard deviation, median (interquartile range), or counts and percentages. Statistical differences between categorical variables were compared using a chi-square test, while continuous variables were compared using a one-way analysis of variance or a Kruskal–Wallis test. Independent associations between RC and depression were examined using a logistic regression model and presented as adjusted odds ratios (OR) and 95% confidence intervals (CI). Based on published literature and clinical expertise, potential covariates were incorporated into multivariable models (16, 22). Model 1 represented univariate logistic regression analysis, Model 2 was adjusted for age and gender, and Model 3 was adjusted for age, gender, BMI, residence, education level, marital status, smoking and drinking, diabetes, hypertension, dyslipidemia, chronic comorbidities, overall health, cognitive function, and CESD-10 score at baseline. The potential nonlinear relationship between RC levels and depression was examined using restricted cubic splines. Knots were placed at the 25th, 50th (median), and 75th percentiles of the RC levels, and the RC variable was analyzed in its original scale without transformation.

Subgroup analyses were used to assess the relationship between RC and depression risk across various subgroups, including age (45–60 years and ≥60 years), gender (male and female), BMI (<28 kg/m^2^ and ≥28 kg/m^2^), diabetes (yes and no), and dyslipidemia (yes and no). All statistical analyses were done on R version 4.2.2 (R Foundation for Statistical Computing, Vienna, Austria). Based on two-sided tests, P < 0.05 indicated statistically significant differences.

## 3 Results

### 3.1 Baseline characteristics

This study involved 7,305 participants (males: 3,632, females: 3,673) with a mean age of 60.0 ± 9.5 years. In the overall population, the mean RC value was 18.48 ± 5.79 mg/dl. A summary of the study participants’ baseline characteristics, based on RC quartile levels is shown in **Table 1**. Baseline characteristics like age, gender, BMI, education level, chronic diseases, hypertension, diabetes, dyslipidemia, and blood lipid indicators, including triglycerides, TC, LDL-C, and HDL-C differed significantly across the four groups (all P < 0.05). Furthermore, the Q4 group had higher BMI and more individuals with at least one chronic disease, especially hypertension and diabetes.

**Table 1:** Baseline characteristics according to remnant cholesterol quartiles at baseline.

Characteristic	Remnant cholesterol (mg/dl)					P-value
	Overall	Q1 (<10.31)	Q2 (≥10.31, <16.48)	Q3 (≥16.48, <24.24)	Q4 (≥24.24)	
	N= 7,305	N = 1,824	N = 1,828	N = 1,826	N = 1,827	
Gender, n (%)						<0.001
Female	3,673 (50.3)	721 (39.5)	923 (50.5)	992 (54.3)	1,037 (56.8)	
Male	3,632 (49.7)	1103 (60.5)	905 (49.5)	834 (45.7)	790 (43.2)	
Age, years	60.0 (9.5)	59.8 (9.7)	60.5 (9.6)	60.5 (9.5)	59.1 (9.0)	<0.001
BMI, kg/m^2^	24.65 (14.11)	23.39 (10.68)	24.12 (11.49)	24.64 (9.66)	26.47 (21.26)	<0.001
Residence, n (%)						0.189
Rural	3,919 (59.8)	997 (61.9)	962 (58.8)	985 (59.5)	975 (59.0)	
Urban	2,636 (40.2)	613 (38.1)	674 (41.2)	671 (40.5)	678 (41.0)	
Educational, n (%)						<0.001
Illiterate	1,700 (23.2)	388 (21.3)	446 (24.4)	466 (25.5)	400 (21.9)	
Elementary school	3,227 (44.2)	874 (47.9)	817 (44.7)	764 (41.9)	772 (42.3)	
Middle school and above	2,378 (32.6)	562 (30.8)	565 (30.9)	596 (32.6)	655 (35.9)	
Health, n (%)						0.092
Poor	188 (2.6)	38 (2.1)	54 (3.0)	50 (2.8)	46 (2.6)	
Fair	953 (13.3)	205 (11.5)	245 (13.6)	253 (14.1)	250 (13.9)	
Good	4,024 (56.0)	1,008 (56.4)	1,021 (56.7)	1,012 (56.4)	983 (54.6)	
Very good and above	2,020 (28.1)	536 (30.0)	481 (26.7)	480 (26.7)	523 (29.0)	
Marital status, n (%)						0.132
Single	1,081 (14.8)	292 (16.0)	283 (15.5)	258 (14.1)	248 (13.6)	
Married	6,224 (85.2)	1,532 (84.0)	1,545 (84.5)	1,568 (85.9)	1,579 (86.4)	
Smoking, n (%)	2,100 (28.7)	526 (28.8)	523 (28.6)	538 (29.5)	513 (28.1)	0.087
Drinking, n (%)	2,734 (37.4)	713 (39.0)	670 (36.7)	701 (38.4)	650 (35.6)	0.056
Chronic diseases, n (%)						<0.001
0	4,999 (68.4)	1,331 (73.0)	1,253 (68.5)	1,220 (66.8)	1,195 (65.4)	
1	1,196 (16.4)	253 (13.9)	309 (16.9)	301 (16.5)	333 (18.2)	
≥2	1,110 (15.2)	240 (13.1)	266 (14.6)	305 (16.7)	299 (16.4)	
Diabetes, n (%)	796 (10.9)	174 (9.5)	188 (10.3)	198 (10.8)	236 (12.9)	<0.001
Hypertension, n (%)	1,468 (20.1)	337 (18.5)	373 (20.4)	379 (20.8)	379 (20.7)	<0.001
Dyslipidemia, n (%)	876 (12.0)	161 (8.8)	179 (9.8)	259 (14.2)	277 (15.2)	<0.001
RC, mg/dl	18.48 (5.79)	6.15 (2.94)	13.20 (3.81)	20.37 (3.31)	34.19 (7.39)	<0.001
TG, mg/dl	118.91 (23.78)	75.77 (20.36)	97.64 (25.79)	116.86 (21.77)	189.31 (27.46)	<0.001
TC, mg/dl	183.84 (25.46)	167.42 (23.60)	179.05 (24.04)	188.30 (26.89)	200.57 (30.29)	<0.001
LDL-C, mg/dl	109.89 (28.04)	114.56 (25.16)	107.93 (25.65)	103.14 (26.17)	103.93 (27.90)	0.019
HDL-C, mg/dl	58.24 (11.47)	54.71 (11.86)	53.92 (11.39)	50.45 (11.08)	45.90 (9.20)	<0.001
CESD-10 score in 2015	6.31 (3.56)	5.79 (3.45)	5.88 (3.48)	6.42 (3.65)	7.10 (3.73)	<0.001
CESD-10 score in 2018	7.85 (5.23)	6.27 (4.86)	6.79 (5.62)	8.36 (4.93)	9.99 (4.65)	<0.001

Continuous variables were shown in mean (SD) and categorical variables were shown in percentages.

BMI, body mass index; CESD-10, 10-item Center for Epidemiological Studies Depression Scale; HDL-C, high-density lipoprotein cholesterol; LDL-C, low-density lipoprotein cholesterol; RC, remnant cholesterol; TC, total cholesterol; TG, triglyceride.

### 3.2 Association between RC and depression

**Figure 2:**
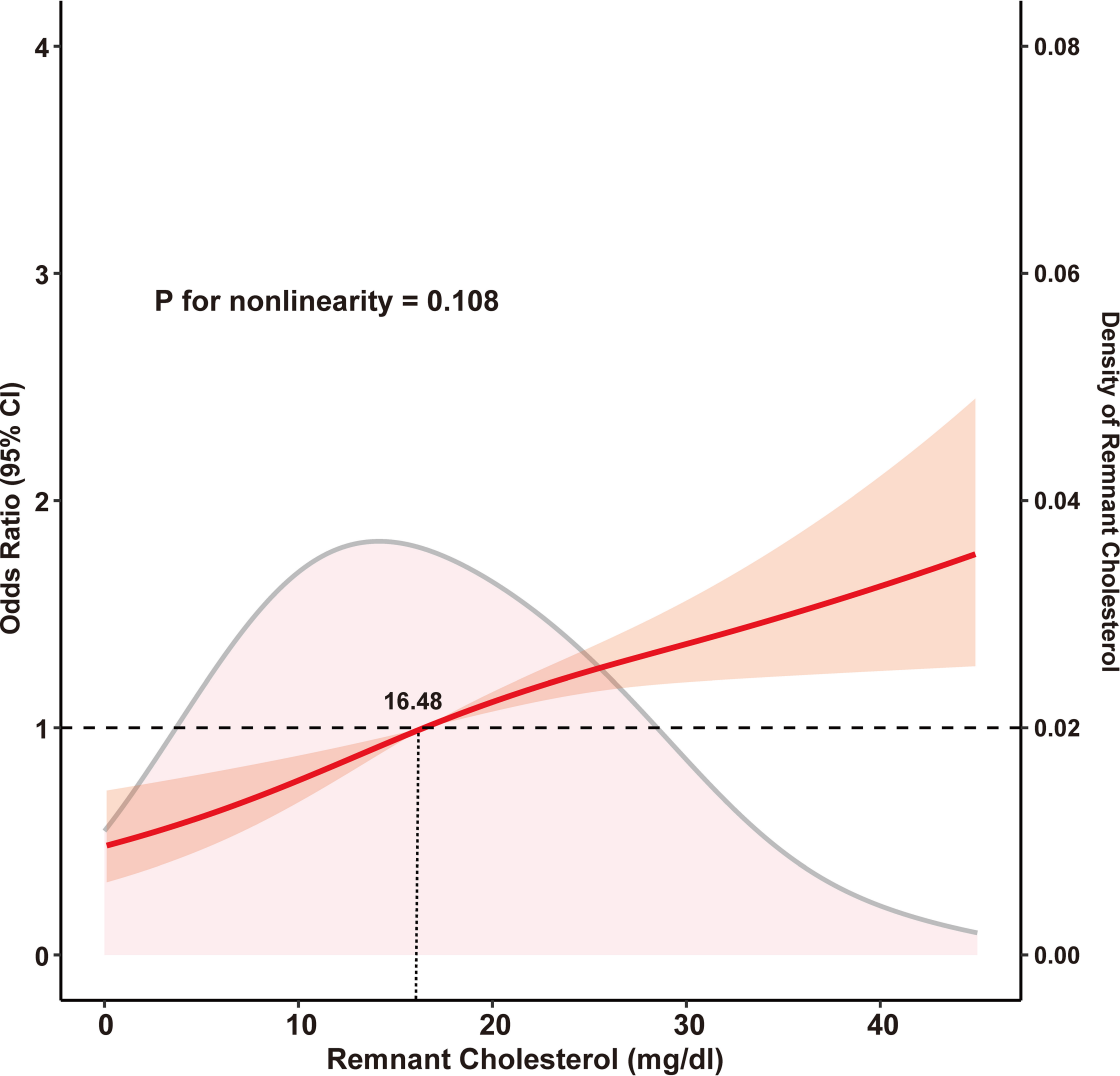
Restricted cubic spline analysis with multivariate-adjusted associations between remnant cholesterol and depression. Age, gender, body mass index, residence, educational level, smoking, drinking, diabetes, hypertension, dyslipidemia, chronic comorbidities, health status, and cognitive function at baseline were adjusted.

In 2015, depression assessments revealed a mean score of 6.31 ± 3.56, which, notably, increased to 7.85 ± 5.23 in 2018. Adjusting for confounding variables revealed a significant relationship between RC quartiles and depression. When compared with participants in Q1, those in Q3 and Q4 exhibited a higher depression risk (OR: 1.75, 95% CI: 1.29–2.39, P < 0.001 and OR: 2.68, 95% CI: 1.96–3.68, P < 0.001, respectively). However, in the Q2 group, the association between RC and depression was not statistically significant in the 2018 follow-up (P > 0.05). Similarly, continuous analyses revealed that each standard deviation increase in RC levels was associated with a 13% increase in depression risk (**Table 2**). Furthermore, multivariable-adjusted restricted cubic splines revealed a linear dose-response association between RC levels and depression (nonlinearity: P = 0.108, **Figure 2**).

**Table 2:** The association between remnant cholesterol and the risk of depression.

	Model 1 ^a^		Model 2 ^b^		Model 3 ^c^	
	OR (95% CI)	P Value	OR (95% CI)	P Value	OR (95% CI)	P Value
Per 0.1 Unit increase	1.69 (1.51-1.91)	<0.001	1.35 (1.20-1.64)	<0.001	1.16 (1.03-1.37)	<0.001
Quartile 1	Ref.		Ref.		Ref.	
Quartile 2	1.31 (1.09-1.58)	<0.001	1.24 (1.03-1.50)	0.022	1.01 (0.73-1.41)	0.936
Quartile 3	1.86 (1.56-2.22)	<0.001	1.73 (1.45-2.08)	<0.001	1.79 (1.31-2.46)	<0.001
Quartile 4	3.17 (2.68-3.77)	<0.001	2.94 (2.48-3.49)	<0.001	2.68 (1.96-3.68)	<0.001
P for trend		0.001		<0.001		<0.001

OR, odds ratios; CI, confidence intervals; CESD-10, 10-item Center for Epidemiological Studies Depression Scale.

a unadjusted.

b adjusted for age, gender.

c adjusted for age, gender, body mass index, residence, educational level, marital status, smoking, drinking, diabetes, hypertension, dyslipidemia, chronic comorbidities, health status, cognitive function, CESD-10 score at baseline.

### 3.3 Subgroup analyses

**Figure 3:**
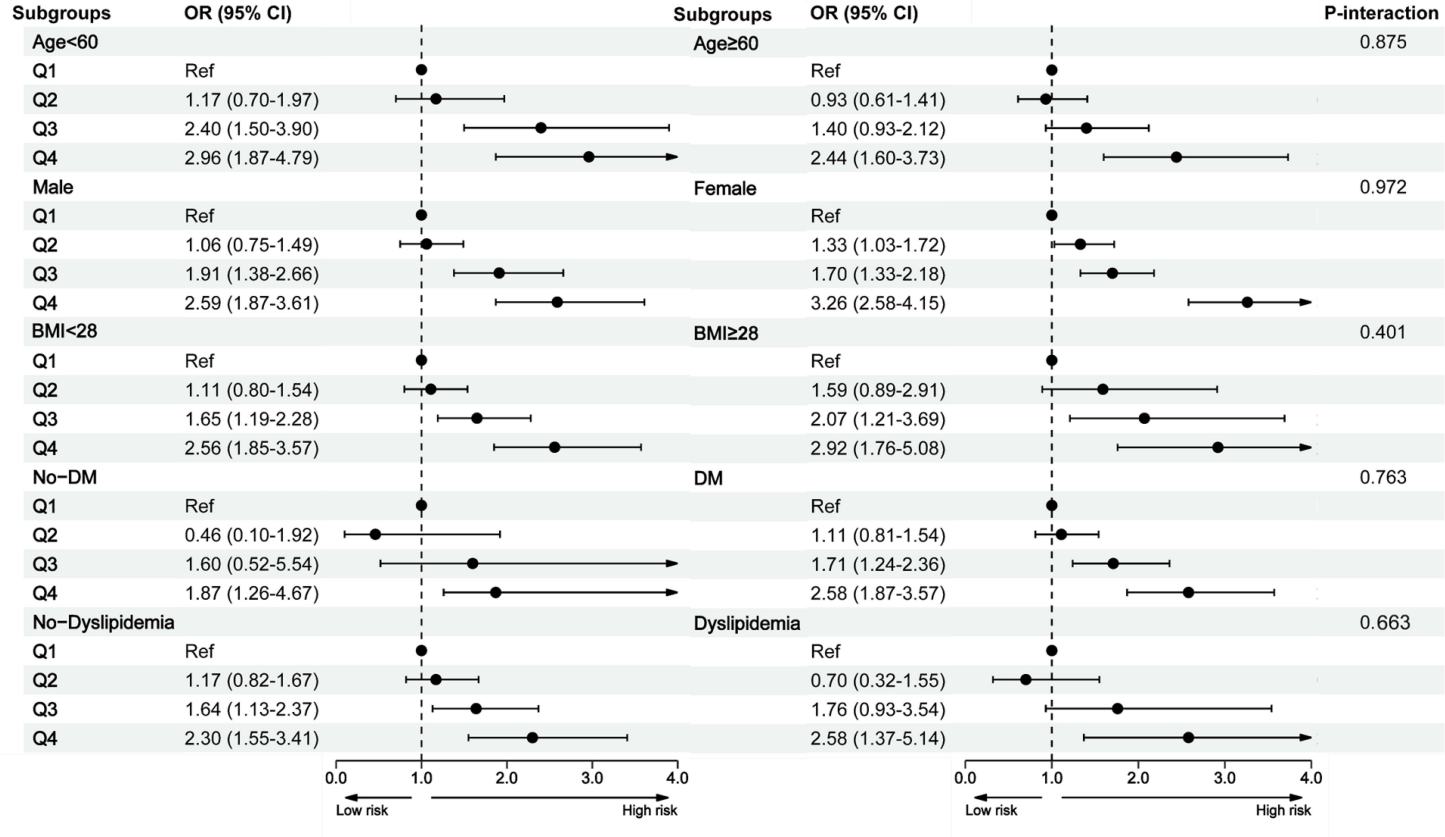
Subgroup analysis between the remnant cholesterol and depression. BMI, body mass index; CI, confidence intervals; DM, diabetes; OR, odds ratios.

The analyses of various stratified subgroups, such as age, gender, BMI, diabetes, and dyslipidemia, revealed consistent outcomes, without significant interaction effects (interaction: all P > 0.05). For all subgroups, being in Q4 (highest RC quartile) exhibited a significant association with an increased depression risk (**Figure 3**).

## 4 Discussion

Our findings offer new evidence of an association between RC levels and depression in middle-aged and older Chinese individuals. Our analyses revealed a positive correlation between elevated RC levels and depression, as well as a linear relationship between RC and depression risk. Moreover, subgroup analysis provided support for a stable association between RC and depression. These findings highlight RC as a potential, important predictor of depression, as well as a depression prevention target.

Previous studies of the relationship between blood lipid biomarkers and depression have suggested that depression may be associated with elevated TC^4^ and triglycerides levels^5^, as well as reduced LDL-C^6^, HDL-C^5^, and omega-3 polyunsaturated fatty acids levels^7^. Notably, when compared with the healthy control group, HDL-C levels were found to be significantly higher in patients with severe depression (23). Moreover, a meta-analysis of the link between lipid levels and depression revealed a trend toward a positive correlation between HDL-C and depression, which was statistically significant in females only (4). However, the association between traditional blood lipid biomarkers and depression is being debated. Although some findings indicate that in older women, lower HDL-C levels are associated with depressive symptoms (24), other studies have found no significant association between depression and LDL-C^4,8^ or TC levels^8,9^, or have observed associations in certain groups only, such as older males in some countries (e.g., Finland) or in young females, where an association between LDL-C and depression has been noted (6). These inconsistent findings limit the clinical applicability of lipid biomarkers and highlight the need for novel lipid parameters for predicting and intervening against depression.

In fasting and non-fasting conditions, RC, the cholesterol content of triglyceride-rich lipoproteins, is made up of very low-density lipoproteins, intermediate-density lipoproteins, and chylomicron remnants (10). An important cholesterol component (25), RC accounts for almost half of atherosclerotic plaque cholesterol content (26). Mounting evidence indicates that RC is associated with various diseases (27–30). Using a cohort of 19,650 individuals in the National Health and Nutrition Examination Survey (NHANES) database, Zhang et al. identified a positive correlation between RC and cardiovascular mortality (31). Moreover, studies of the relationship between RC and renal function have revealed a negative correlation between RC levels and the estimated glomerular filtration rate (32). Furthermore, studies involving 60-year-old individuals suggested that there is a negative correlation between RC and memory function and language acquisition, suggesting that in older adults, lower RC levels may prevent cognitive impairment (33). Although increasing evidence suggests that RC may be involved in other diseases, its association with depression has not been adequately elucidated. Although a recent cross-sectional analysis of data from the NHANES database suggested a positive relationship between RC and depression, it mainly focused on the United States general adult population and had a cross-sectional design. To build upon these findings, we conducted a follow-up observational study of middle-aged and older individuals in China’s CHARLS database. Our findings revealed a significant positive correlation between RC and depression in Chinese individuals aged ≥45 years, and subgroup analysis based on various factors, including age and gender, obtained consistent results. Therefore, RC may serve as a novel lipid metabolic biomarker of depression. Notably, our gender-stratified subgroup analysis revealed that higher RC levels were consistently associated with an increased risk of depression in both males and females. These findings indicate the potential of RC as a universal marker for depression risk assessment, rather than being limited to specific gender populations.

The mechanistic link between RC and depression is complex and poorly elucidated, and there are several proposed mechanisms for this relationship. Firstly, RC has been associated with low-grade inflammation (34) and endothelial dysfunction (35), well-known contributors to arterial stiffness (36), which can cause cerebral microvascular dysfunction (37). Moreover, previous studies have associated greater arterial stiffness with an increased risk of developing depressive symptoms (38). Secondly, elevated serum RC levels can enhance arterial wall permeability, which makes macrophages capture and absorb RC more readily than LDL-C, thereby accelerating foam cell formation (39). Foam cells express interleukin-6, and circulating interleukin-6 can stimulate the hypothalamic–pituitary–adrenal axis (40), and hypothalamic axis changes are implicated in depression and cognitive impairment (41–43). Finally, RC may contribute to depression by generating cytokines, which act on neural cells. By crossing the blood–brain barrier or via other entry pathways, peripheral cytokines can directly impact neurons and support cells (44).

Depression, a prevalent and far-reaching mental disorder, is more prevalent in middle-aged and older individuals. However, various factors, including limited awareness, hinder its early detection and management, which compromises individual health while imposing a significant societal burden, especially considering the limited efficacy of pharmacological depression treatments in this age group. Therefore, early depression prevention is a crucial and pressing need. Based on population-based evidence, we have identified RC levels as a relatively new blood lipid biomarker that correlates positively with depression. Our findings offer valuable insights into depression pathogenesis. Additionally, because RC indicators are easily accessible from routine blood lipid profiles at no additional cost, they have the potential for widespread clinical application in the early detection of individuals with a high depression risk. Furthermore, this highlights RC as a promising therapeutic target against depression.

This study has some limitations. First, it is important to recognize that the study was observational. Although adjustments were made for confounding factors, the potential influence of unmeasured confounding variables may still be present. Secondly, some participants in the cohort were lost to follow-up, and certain data were collected using standardized questionnaires, which may have introduced potential selection bias and recall bias. Furthermore, as the study population is exclusively Chinese adults aged 45 years and older, additional research involving more diverse populations, such as individuals from different age groups or regions, is necessary to further validate the robustness of the findings. Additionally, the study lacks comprehensive information on the use of medications that affect blood lipids. Consequently, our findings’ generalizability may be limited. Therefore, further studies are required to validate our findings.

## 5 Conclusion

This study’s findings indicate that elevated RC levels are associated with an increased depression risk in middle-aged and older individuals, demonstrating a linear dose-response correlation. Prospective studies are required to validate this study’s findings and to explore strategies for optimizing RC therapeutic efficacy in patients with depression.

## Data Availability

Publicly available datasets were analyzed in this study. This data can be found here: http://charls.pku.edu.cn/en.
