# Supplementary material for: Association between remnant cholesterol and depression in middle-aged and older Chinese adults: a population-based cohort study
Source: Front Endocrinol (Lausanne). 2025 Feb 3;16:1456370. doi: 10.3389/fendo.2025.1456370 (PMC11830595; doi:10.3389/fendo.2025.1456370)
Supplement: Supplementary file 1 [file Table1.docx]

**Additional Table 1 Baseline characteristics of participants aged 45 years and older: included vs. excluded Due to Missing CESD Scores in 2018**

| **Characteristic** | **Included Participants** | **Excluded Participants** | **P-value** |
| --- | --- | --- | --- |
|  | **N= 7,305** | **N = 2,331** |  |
| Female, n (%) | 3,673 (50.3) | 1,123 (48.2) | 0.081 |
| Age, years | 60.0 (9.5) | 59.1 (9.3) | 0.709 |
| BMI, kg/m^2^ | 24.65 (14.11) | 23.39 (13.21) | 0.041 |
| **Residence, n (%)** |  |  | 0.089 |
| Rural | 3,919 (59.8) | 1,153 (57.6) |  |
| Urban | 2,636 (40.2) | 848 (42.4) |  |
| **Educational, n (%)** |  |  | 0.634 |
| Illiterate | 1,700 (23.2) | 531 (22.8) |  |
| Elementary school | 3,227 (44.2) | 1,056 (45.3) |  |
| Middle school and above | 2,378 (32.6) | 744 (31.9) |  |
| **Health, n (%)** |  |  | 0.005 |
| Poor | 188 (2.6) | 91 (3.9) |  |
| Fair | 953 (13.3) | 329 (14.1) |  |
| Good | 4,024 (56.0) | 1,294 (55.5) |  |
| Very good and above | 2,020 (28.1) | 617 (26.5) |  |
| **Marital status, n (%)** |  |  | 0.200 |
| Single | 1,081 (14.8) | 371 (15.9) |  |
| Married | 6,224 (85.2) | 1,960 (84.1) |  |
| Smoking, n (%) | 2,100 (28.7) | 683 (29.3) | 0.624 |
| Drinking, n (%) | 2,734 (37.4) | 921 (39.5) | 0.075 |
| **Chronic diseases, n (%)** |  |  | 0.217 |
| 0 | 4,999 (68.4) | 1,550 (66.5) |  |
| 1 | 1,196 (16.4) | 403 (17.3) |  |
| ≥2 | 1,110 (15.2) | 378 (16.2) |  |
| Diabetes, n (%) | 796 (10.9) | 261 (11.2) | 0.718 |
| Hypertension, n (%) | 1,468 (20.1) | 501 (21.5) | 0.153 |
| Dyslipidemia, n (%) | 876 (12.0) | 303 (13.0) | 0.209 |
| TG, mg/dl | 118.91 (23.78) | 123.82 (23.99) | 0.063 |
| TC, mg/dl | 183.84 (25.46) | 186.71 (26.03) | 0.079 |
| LDL-C, mg/dl | 109.89 (28.04) | 112.33 (28.74) | 0.055 |
| HDL-C, mg/dl | 58.24 (11.47) | 56.62 (10.88) | 0.103 |
| CESD score in 2015 | 6.31 (3.56) | 6.61 (3.77) | 0.087 |

Continuous variables were shown in mean (SD) and categorical variables were shown in percentages.

**Abbreviation:** BMI = body mass index; CESD: Center for Epidemiologic Studies Depression Scale; HDL-C: high-density lipoprotein cholesterol; LDL-C: low-density lipoprotein cholesterol; RC: remnant cholesterol; TC: total cholesterol; TG: triglyceride.
